# Using Music to Promote Hong Kong Young People’s Emotion Regulation and Reduce Their Mood Symptoms and Loneliness: Protocol for a Pilot Randomized Controlled Trial

**DOI:** 10.2196/67764

**Published:** 2025-04-16

**Authors:** Yuan Cao, Yuanxin Shi, Debbie Chi Wing Low, Daniel T L Shek, David H K Shum, Radhika Tanksale, Genevieve Dingle

**Affiliations:** 1 Department of Social Work and Social Administration The University of Hong Kong Pok Fu Lam China (Hong Kong); 2 Department of Applied Social Sciences The Hong Kong Polytechnic University Kowloon China (Hong Kong); 3 School of Psychology University of Queensland Brisbane Australia

**Keywords:** youth, adolescents, adolescence, teens, teenagers, music, moods, mood symptoms, loneliness, emotion regulation, emotions, Hong Kong, mental health, mental illnesses, mental disorders, randomized controlled trial, RCTs, protocol

## Abstract

**Background:**

Mental health needs in the community surged during the pandemic, with concerning reports of increased negative mood symptoms among youth. At the same time, preventive psychoeducational interventions were insufficient within frontline youth mental health services in Hong Kong, and research specifically addressing youth loneliness remained limited on an international scale. Given the association between loneliness and other mental health symptoms, psychoeducational programs that empower adolescents to cope with emotions may help address both the research gap and local demand. As such, Tuned In, a previously validated intervention program originally developed in Australia, was introduced to the local context. Cultural adaptations and an added focus on loneliness were incorporated into the project to enhance its acceptability and test its effectiveness.

**Objective:**

This study aims to evaluate an adapted version of the Tuned In music-based psychoeducation program, designed to reduce loneliness, depression, and anxiety symptoms among young people in Hong Kong by enhancing their emotion regulation skills.

**Methods:**

Participants aged 16-19 years will be randomly assigned to either the experimental or control group. The experimental group will receive an online, group-based psychoeducation program focused on emotion recognition and management, delivered weekly over 4 consecutive weeks. The intervention is grounded in Russell’s emotion circumplex model and music psychology, and program content included: The 2D model and characteristics of emotions from different quadrants (session 1); happiness and loneliness (session 2); high-arousal and negative-valence emotions, for example, stress and anxiety (sessions 3); and anxiety, perfectionism, and a celebration of achievement (session 4). Both therapist- and participant-selected music will be used in the intervention to provide a rich repertoire for group discussion, psychoeducation, reflection, and the practice of social skills. The main outcome measures will be assessed using the Emotion Regulation Questionnaire, the Difficulties in Emotion Regulation Scale, the Depression Anxiety Stress Scale, and the De Jong Gierveld Loneliness Scale. Feedback on the project arrangement will be gathered through qualitative input. A mixed methods analysis will be conducted following data collection.

**Results:**

The project was successfully funded in February 2023 by the Health and Medical Research Fund in Hong Kong and commenced in August 2023. As of September 16, 2024, a total of 316 completed questionnaires had been received through Qualtrics for screening purposes, with 89 participants deemed eligible for the program. The project is scheduled to conclude in August 2025, with results to be published thereafter.

**Conclusions:**

Participants are expected to show improvements in emotion regulation, along with reductions in mood symptoms and loneliness, following the intervention.

**Trial Registration:**

ClinicalTrials.gov NCT06147297; https://clinicaltrials.gov/study/NCT06147297

**International Registered Report Identifier (IRRID):**

DERR1-10.2196/67764

## Introduction

### Significance of Study

Youth mental health is a significant concern in Hong Kong. Studies have reported that the prevalence of depression and anxiety among adolescents is approximately 11.3% locally [1]. Loneliness is also widespread among young people in Hong Kong, occurring at a higher rate than that reported for their counterparts in North America [2]. Around 39% of young people in Hong Kong reported feeling lonely—an increase of 55% from 2012 to 2018 [3]. Furthermore, the prevalence of loneliness rose to 47% among adolescents and young adults in 2020 due to the pandemic [4]. A recent survey found that loneliness was associated with symptoms of depression, anxiety, and stress, even after controlling for demographic factors and views on the COVID-19 pandemic [2]. These negative emotional states have also been shown to be linked to other issues, such as substance use [5]. The connection between negative experiences, low mood, and substance use problems may be partly explained by a lack of emotion regulation skills. Emotion regulation involves the ability to manage emotions within a social context, which includes expressing emotions appropriately and with suitable intensity [6]. The Transdiagnostic Emotion Vulnerabilities Model proposes that emotional vulnerability is a core transdiagnostic factor underlying major depression, anxiety, and tobacco smoking [7]. In particular, young people may believe that unhealthy coping mechanisms, such as smoking, help them to relax. Because of this pairing of mental distress and poor health habits, they are likely to engage in—or continue—smoking during times of distress [5]. By contrast, a meta-analysis by Schafer et al [8] found that healthy coping during distress was associated with reduced depressive and anxious symptoms (*r* ranging from –0.29 to –0.50) among adolescents and young adults.

Given the significant role that emotion regulation plays in promoting youth mental health, it is desirable for preventative interventions to equip young people with age-appropriate, healthy coping strategies that serve as protective factors against long-term emotional difficulties, health problems, addiction, or substance abuse. Unfortunately, few such interventions have been validated by research in Hong Kong [9]. The proposed pilot trial will evaluate the effectiveness of a music-based prevention program in reducing mood problems among young people by enhancing their emotion regulation skills. This project is supported by a seed grant to conduct a small-scale trial in preparation for future large-scale research.

To evaluate the validity and effectiveness of the project in the Hong Kong context, the following hypotheses will be tested:

- The emotion regulation skills of adolescents and young adults will be enhanced following participation in the music-based program (primary outcome).
- The depression and anxiety symptoms of adolescents and young adults will be reduced following participation in the music-based program (secondary outcome).
- The sense of loneliness experienced by adolescents and young adults will be reduced following participation in the music-based program (secondary outcome).

### Definition of Terms of Music Interventions

Music, defined by The New Penguin English Dictionary as “any vocal, instrumental or mechanical sounds that have rhythm, melody or harmony” [10], can influence emotions in many ways. As such, music is often perceived as the language of emotion [11]. Distinct from the term song, which refers to a short composition combining both words and music, music is considered a hypernym of song. In other words, music is a broader term that encompasses a range of activities—from listening to background music to engaging in composition and improvisation [12]—whereas song specifically refers to music with lyrics that can be sung [13]. This broader definition of music is adopted by music therapists to allow greater flexibility in the use of music or sound during interventions, enabling them to meet clients where they are in the present moment [14].

Music interventions are broadly classified into 2 types: active and receptive [15]. In active interventions, clients participate in the process of music production. By contrast, receptive interventions involve clients receiving the music experience, with listening focused on the intellectual, emotional, or other aspects of the music [15,16]. Previous studies on group music interventions for young people have not found significant differences in treatment outcomes between the 2 approaches [17].

### Music and Emotions

The activation of the limbic system—responsible for processing emotions—when the ears perceive music provides a biological foundation for music’s influence on emotions [18]. The emotions experienced by listeners result from an interplay of multiple factors, including elements of the music itself, the performance, and the surrounding environment. For example, structural features of music such as tempo and melody are known to influence emotional responses. It is widely accepted that tempo can directly affect the pleasantness of emotions and that melodies tend to be higher in pitch in happy music compared with sad music [19]. In addition to musical structural features, song lyrics are often used as a springboard for discussing life events that evoke emotions during interventions [16]. Lyrics play a vital role in eliciting emotions, particularly sadness [20]. Previous research has also found that engaging with self-selected music can foster a sense of connectedness [21].

### Music Interventions, Youth Emotion Regulation, and Tuned In

Listening to music is an effective emotion regulation strategy [22-24], and existing literature supports its use in reducing symptoms of depression and anxiety. One study examining the use of online group music therapy to manage anxiety and stress among university students confirmed the effectiveness of this type of intervention [17].

Tuned In, a preventative program developed by 1 of the coinvestigators to address mild to moderate levels of psychological distress, has demonstrated positive psychological outcomes in both adolescents [25,26] and young adults in Australia [6]. A related intervention, Smoke into Sound, was found to be more effective in reducing cigarette cravings than the gold standard of cognitive behavioral therapy delivered via telephone, as shown in a randomized controlled trial (RCT) [27].

During the COVID-19 pandemic, young adults rated listening to music as the most effective coping mechanism [24]. The Tuned In program is based on Russell’s [28] circumplex model of emotion, which categorizes emotions along 2 dimensions: valence and arousal. The theoretical foundation linking music and emotion in the Tuned In program draws from this 2D model [28,29], in which emotions are positioned along an arousal axis (ranging from high to low energy) and a valence axis (ranging from pleasant to unpleasant). It also incorporates the BRECVEMA (brain stem reflexes, rhythmic entrainment, evaluative conditioning, contagion [emotional contagion], visual imagery, episodic memory, musical expectancy, and aesthetic judgment) theory of music and emotion [30], which identifies mechanisms such as lyrical meaning, personal memories associated with music, and imagery evoked by music as key factors influencing emotional responses to listening. Although not explicitly stated in the program materials, this theoretical background is embedded in the content and activities—such as lyric analysis and imagery drawing tasks conducted while listening to music.

Through psychoeducation and experiential activities designed to explore and make meaning of emotions—such as locating emotions in the body, lyric analysis, drawing imagery while listening to music, and group discussions—participants in the program first learn to categorize their emotions in terms of intensity (ie, arousal level) and positivity (ie, valence). They then reflect on and develop a personalized list of songs they relate to, using these songs to increase or decrease the intensity and positivity of their emotions. In other words, participants practice monitoring their emotional states and using music to help modify how they feel.

In addition, music can enhance social connectedness—and potentially foster actual social connections—when shared with others [12]. Therefore, music serves as a universal and comprehensive tool for promoting emotion regulation and reducing feelings of loneliness, as well as depressive and anxious symptoms [24,25,31-33]. Furthermore, emerging local evidence supports the positive role of music listening as a promising strategy for enhancing mental health among adolescents in Hong Kong. In a large-scale cross-sectional survey, Leung and Cheung [34] found that music listening contributed to psychological well-being among young people by enhancing emotional awareness.

### Music Interventions, Youth Loneliness, and Tuned In

In addition to depression and anxiety, loneliness is a socioemotional state that has attracted increasing research attention [35]. In 2019, 22% of young Chinese people reported experiencing feelings of loneliness [36]. Recent data also suggest that loneliness is associated with poor health habits—such as smoking—among young Chinese individuals [36].

Although lonely young people may have friends and families, everyday interactions often provide limited opportunities for deep connection and personal sharing within a trusting environment for this age group [37]. This is echoed by recent research findings showing that 76% of lonely adolescents and young adults feel they are not well understood by others—a situation that could potentially be addressed through the empathic effects of music [21,31].

Feelings of empathy or being understood can arise not only through human interactions but also through listening to comforting music, suggesting a promising mechanism for reducing loneliness in young people [21]. Schafer and colleagues [21] found that listening to self-selected music improved participants’ self-reported mood following a sad mood induction, regardless of the type of music chosen. They proposed that self-selected (ie, familiar) music acts as a surrogate listener, helping individuals feel that their emotions are being “heard,” thereby providing a comforting effect. These soothing effects of music could be leveraged to address a key issue underlying loneliness among young people.

However, there is limited research on interventions aimed at reducing loneliness among young people in Hong Kong. This scarcity is also evident in the broader literature. A recent meta-analysis and systematic review found that certain group-based psychological interventions can be effective in reducing loneliness [38,39].

One psychoeducation group intervention found no significant decrease in loneliness at follow-up [40], while other interventions—such as online dancing and various psychoeducation-based group programs—reported reductions in loneliness [41-43]. These mixed results highlight the need for more promising and effective programs to reduce loneliness, both locally and within the broader research literature.

Nevertheless, there is preliminary evidence that the Tuned In program has positive effects in reducing loneliness among young people. Coinvestigator GD and her team (email communication, February 18, 2025) completed a study applying the original program in a passive control design with 35 English-fluent international students in Australia. They found a significant interaction between time and condition, resulting in a reduction in self-reported loneliness among participants—with a moderate effect size (η^2^_p_=0.12)—compared with the waitlist group. This overall positive effect is attributed to the synergy of the program’s core components: psychoeducation, group work, and music. Each serves as an essential therapeutic element, and together they form the comprehensive Tuned In intervention.

Undeniably, adopting an active control would be more ideal. However, due to limited resources and the need to maintain fidelity to the original program, a waitlist control design was chosen for practical reasons. The primary aim of this study is to examine whether this combination of elements can produce desirable outcomes in the Hong Kong context. Investigating the specific role and contribution of each component, as well as comparing the effectiveness of this combination with other types of interventions, represents a valuable direction for future research.

### Theoretical Framework for Tuned In

The Tuned In program was developed as an alternative to cognitive behavioral therapy and is grounded in emotion theory and music psychology research on the links between music listening and emotional responses [25-27]. The 4 sessions primarily aim to not only address negative emotions—such as depression, loneliness, anxiety, and anger—but also explore ways to enhance positive emotions or states such as motivation, happiness, and calmness. Each session begins with the facilitator describing the components and functions of emotions (eg, physiological responses and common thoughts), followed by participant sharing and group discussion about songs they currently listen to—or could listen to—that elicit or help regulate those emotions.

As engagement in music does not require participants to have prior knowledge or skills in music [17], a receptive intervention has been chosen for the current project. According to Juslin’s BRECVEMA model [30], the meaning of lyrics is one of several mechanisms that link music listening with emotional responses. The model identifies multiple pathways through which a musical event can influence emotions, including brain stem reflex, rhythmic entrainment, evaluative conditioning, emotional contagion, visual imagery, episodic memory, musical expectancy, and aesthetic judgment [30].

Although some experimental evidence suggests that melody has a stronger influence than lyrics on emotional responses to music [44], this effect is highly individualized—some people focus more on melody, while others are more affected by lyrics. Therefore, both instrumental music and songs will be used in this project. In alignment with both the original program and guidelines for receptive music interventions, instrumental music may be used for imaginal and somatic listening, with the flexibility to adjust based on participants’ emotional states and feedback. Songs, by contrast, will be used for lyric discussions. Given that the current project is psychoeducational, with music serving as a tool for relationship building and emotional regulation, the inclusion of both songs and instrumental music allows for greater acceptance of diverse musical styles and gives participants the freedom to share and appreciate various kinds of music. Discussions about song components (eg, lyrics) can also deepen participants’ understanding of the physiological and cognitive aspects of different emotions. The intention is that, by the end of the program, participants will be able to assess their emotional valence and arousal, and they will have a personalized playlist they can use to enhance or regulate their emotional states. In other words, they will be “tuned in” to their emotions.

Building on the existing evidence, the proposed study aims to establish an effective music-based program to enhance emotion regulation skills among young people in Hong Kong. This approach complements current practices in the region, which often focus on areas such as self-esteem, resilience, social competence, problem-solving, and communication [1,9]. Notably, the proposed study introduces a new session targeting “loneliness”—an addition to the original Tuned In program implemented in Australia [6]. This new component directly addresses the rising prevalence of loneliness among adolescents in Hong Kong [3,4].

The program will be facilitated by a registered music therapist and a research assistant, both under the supervision of the primary investigator. The research assistant will receive training from the coinvestigator and original developer of Tuned In before delivering the groups, along with ongoing supervision from her. This arrangement ensures the standardized delivery of both the music-based activities and the psychoeducational content. Because of previous COVID-19–related restrictions, the program was initially proposed to be conducted online. However, with the loosening of COVID-19 restrictions, the study has incorporated an introductory session where participants can meet each other and the facilitator in person. This session is intended to build rapport and foster a sense of group identity, which is expected to enhance engagement during the subsequent online group sessions while preserving the flexibility of the program format [45,46].

### Aims and Objectives

The ultimate aim of this study is to test and validate a music-based program designed to improve emotion regulation among adolescents and young adults in Hong Kong, with the broader goal of reducing symptoms of depression, anxiety, and loneliness.

The objective of the proposed project is to evaluate the effects of a music-based program on young people in Hong Kong. The hypotheses to be tested are listed below.

The primary outcome is as follows:

- The emotion regulation skills of adolescents and young adults will be enhanced following participation in the music-based program.

The secondary outcomes are as follows:

- Symptoms of depression and anxiety among adolescents and young adults will be reduced following the program.
- Feelings of loneliness among adolescents and young adults will be reduced following the program.

## Methods

### Ethical Considerations

Ethical approval for the study was obtained from The University of Hong Kong (approval number EA230395) and The Hong Kong Polytechnic University (approval number HSEARS20221024004). Ethical approval was either exempted or not required by other participating universities for the distribution of advertisement materials.

In adherence to the ethical regulations of the university, signed active parental consent will be obtained for participants under the age of 18 years. All participants are required to sign a physical consent form before participating in the intervention. Participation is entirely voluntary, and all participants have the right to withdraw from the project at any time without any negative consequences.

### Study Design

The study adopts a waitlist RCT design. Participants are randomly assigned to either the intervention group, who will receive the program immediately, or the waitlist control group, who will receive the intervention after a 4-week waiting period. The main assessment time points are pre- and postintervention for the intervention group, and baseline and postwaiting period for the control group. Additionally, the control group will be invited to complete an optional postintervention survey following their participation, which will be used for secondary data analysis. The postintervention surveys for both groups will contain identical items to ensure comparability. It is also planned that all participants who complete the intervention will be invited to take part in an optional qualitative interview to gain deeper insights into their experiences using the strategies taught in the program.

Given the project’s strong emphasis on psychoeducation, participants’ application of the strategies taught serves as an indicator of the program’s acceptability. Qualitative inquiry offers valuable insights into the extent to which participants have internalized and consolidated the healthy coping strategies introduced during the intervention. This approach enables exploratory interpretations of the project’s outcomes and helps uncover the nuanced, individualized experiences of participants. Therefore, qualitative research is considered appropriate to capture the subjective and personal dimensions of the intervention’s impact.

Online delivery was chosen in response to the prevailing pandemic conditions at the time. This decision not only aligned with the original program design but also helped overcome geographical barriers. By eliminating distance as a constraint, the online format significantly enhanced the accessibility of the program. While this mode of delivery presented challenges—such as difficulty in maintaining participants’ attention and limited nonverbal communication—it also expanded the potential of digital platforms to support mental health through digital expressive arts and peer support [47]. Moreover, the web-based design increased the mobility and flexibility of the intervention [48]. Previous studies have shown that online group interventions can be effective in reducing mental health symptoms such as depression [43,48], and Draper and Dingle [49] found that virtual groups were effective in addressing psychological needs.

### Material and Personnel Preparation

The group sessions are cofacilitated by 2 individuals: a research assistant with a background in a mental health–related discipline and a registered music therapist. The research assistant takes the lead in delivering the psychoeducational content, while the music therapist facilitates the musical experiential activities and supports group discussions. Both facilitators employ techniques such as encouraging participants to be “good group members” to manage group dynamics effectively. For instance, during ice-breaking activities or group discussions led by the research assistant, the music therapist may participate as though they are a group member. This approach helps to foster rapport and strengthen the connection between the facilitators and participants. The same roles and techniques also apply to the research assistant during music activities. Additionally, the research assistant serves as the main point of contact for participants and is responsible for participant follow-up and distribution of reimbursement. The follow-up, limited to the scope of the project, includes administrative liaison (eg, responding to participant inquiries, delivering materials, arranging logistics) and crisis intervention. Crisis intervention involves providing appropriate community crisis support resources and information and making an informed decision regarding the participant’s eligibility to continue with the group intervention.

To enhance project fidelity, the English manual from the original program was revised and translated into Chinese. The music selections from the original program were also adapted by the music therapist to better suit the local context. The incorporated music in this project is intended to produce equivalent therapeutic effects while maintaining consistency in music type with the original selections. For instance, Shawn Mendes’ *A Little Too Much* [50] from the original program was replaced with Eason Chan’s *Today* [51], which is more culturally relevant and relatable for local youth.

The manuals will be printed and distributed to participants and facilitators before the sessions. In cases where distributing hard copies is not feasible, an electronic version will be provided instead. To enhance readability and engagement during session presentations, PowerPoint (Microsoft Corporation) slides aligned with the manual content will also be used.

A session checklist outlining the key components of each session was prepared for facilitators to log the completion of intervention activities. These entries serve as records to support program fidelity and ensure consistency across sessions.

### Recruitment

The recruitment process began in August 2023 and will continue until August 2025. Various strategies have been implemented to improve the accessibility of project information across all regions of Hong Kong.

Three main recruitment channels have been used:

- Social Media Platforms: project posters, welcome messages, and links to detailed information were posted on Instagram (Meta Platforms, Inc.), Facebook (Meta Platforms, Inc.), and Social Career (Social Career Limited), which are widely used by local youth. Additionally, project details were published on the university website to reach the general public.
- Existing Partnership Networks: Information about the project was shared with 3 local universities, a local integrated children and youth services center, and several secondary schools that either have existing collaborations with the principal investigator (YC) or have expressed partnership interest. These institutions helped disseminate project details to their students or service users (eg, the ELCHK Lutheran Academy).
- Exploratory cold calls and emails: The research assistant reached out to additional secondary schools and integrated children and youth services centers via phone and email. These contacts were selected based on alignment between their service targets, institutional missions, and the goals of the project. Project information and partnership invitations were conveyed either verbally or in writing.

### Randomization and Blinding

Participants will be randomly assigned to either the intervention or waitlist control group using a computer-generated randomization procedure. The Random Allocation Software developed by Dr. Mahmood Saghaei [52] will be used to facilitate this process. Block randomization will be implemented to ensure balanced group sizes, with a maximum of 8 participants per group. Randomization will be carried out by an independent researcher who is not part of the current research team or is listed as an author. The principal investigator remains blinded to group assignments to maintain objectivity and reduce potential bias. Outcome measures are managed by a separate research assistant who is not involved in the delivery of the intervention, ensuring independence and reducing potential bias. Once a sufficient number of eligible participants have been recruited, randomization will be conducted, typically 2 weeks before the intervention start date. Participants will then be informed of their group allocation, along with the schedule of their assigned group sessions, by the designated research assistant. In alignment with the ongoing recruitment process and rolling admission, the intervention will be delivered to participants as they are randomized and assigned to groups.

### Participants

The target population for this study comprises post–high school students and adolescents aged 16-19 years. This age range aligns with international guidelines, such as those from the World Health Organization, which defines adolescence as ranging from 10 to 19 years, and the United Nations, which considers individuals aged 15-24 years as adolescents and young adults. Recruitment materials for the project will be disseminated to students in their final year of high school as well as recent graduates who maintain connections with their schools. Young adults, primarily those in their first year of undergraduate studies (typically aged 17-19 years in the Hong Kong context), will be recruited through posters displayed on university campuses and word-of-mouth referrals. A snowball sampling method will also be used to expand participant recruitment. Consistent with the approach used by Dingle and Fay [6], this study will include youth who self-report experiencing symptoms of low mood, anxiety, or loneliness.

Inclusion criteria for participation are (1) self-reported difficulties with low mood, anxiety, or loneliness; and (2) a score of 3 or above on the 12-item General Health Questionnaire (GHQ-12), indicating the presence of psychological distress. Exclusion criteria are the presence of severe mental health symptoms, such as self-harm or suicidal ideation, as the Tuned In program has not yet been trialed with individuals experiencing more acute mental health conditions.

Interested participants who do not meet the inclusion criteria will be provided with an information sheet about community counseling services. Eligible participants who confirm their enrollment will be randomized.

### Sample Size Calculation

Dingle and Fay [6] found a medium effect size (η^2^_p_=0.076) for improved emotion regulation after the music intervention with young adults, compared with the control group. Based on this medium effect size, at least 98 participants are needed to detect a significant group × time interaction effect (*P*<.05). However, based on previous trials, some dropout is expected after the intervention has commenced. The estimated attrition rate is 15%. Besides, considering that partnerships with middle schools have only recently begun to develop, while many university students have already expressed interest in committing to the project, we expect to recruit more university participants. Therefore, we aim to recruit over 100 participants for the randomization stage, including around 50 adolescents and 65 young adults, to account for possible dropouts before the intervention begins.

### Contingency Plan

Based on prior trials of Tuned In, we expect a high retention rate among youth [6]. However, some attrition may occur among adolescents [25]. Even with that attrition rate, we anticipate retaining at least 30 adolescents at the postintervention stage, providing sufficient data from this pilot study to inform future large-scale trials. In the event of significant challenges in recruiting adolescents, our backup plan is to recruit additional youth from the university community for this trial.

Because of the online mode of intervention, the study design minimizes potential disruptions arising from any local changes in the COVID-19 pandemic situation. If difficulties or delays in participant recruitment arise, we will place advertisements on social media as an additional recruitment channel.

Considering the risk of potential emotional disturbances, a page listing several 24-hour community crisis support hotlines has been incorporated into the manual to provide participants in need with an additional channel to external resources. Alongside the distribution of these resources, the research assistant will also give instructions on how to use them during the group contracting phase and explicitly encourage participants to seek help and notify the research assistant immediately if they experience emotional distress during the session.

In situations where psychological distress is identified either by a facilitator or self-reported by a participant, the research assistant will separate the individual from the rest of the group, while the music therapist continues to lead the remaining activities. The research assistant will provide immediate emotional support and conduct a timely risk assessment, and the principal investigator will be informed of the situation. Based on the participant’s condition, appropriate referrals will be made as necessary.

The research assistant will follow up with individuals who disclose intentions or actions related to self-harm, suicide, harm to others, or breaches of the law, and will discuss with them the appropriateness of continuing in the project or the available options for discontinuation.

### Description of the Intervention

The project consists of 5 weekly sessions: 1 introductory (preassessment) session followed by 4 psychoeducational sessions. Each session lasts approximately 60-90 minutes.

In the introduction session, participants sign the informed consent forms after being informed about the program’s content and expectations by the main group facilitator (ie, the research assistant). For participants under 18 years of age, parental consent will also be obtained during the session. Following the signing of consent, the baseline survey will be distributed and collected. Participants’ demographic information will be gathered through the survey. This includes, but is not limited to, age, gender, education level, household income, region of residence, living arrangement, and employment status.

Each week, participants will receive WhatsApp (Meta Platforms, Inc.) text message reminders about upcoming sessions and other important announcements.

The psychoeducational sessions differ in theme but follow a similar structure. Session 1 focuses on emotions located in various quadrants of the 2D model, such as high-arousal and negative-valence emotions. Session 2 explores happiness and loneliness, with the new component of loneliness added to this session. Sessions 3 and 4 address high-arousal emotions, such as stress and anxiety. An example session plan is provided in Multimedia Appendix 1 for reference (Table 1).

**Table 1:** Summary of session content.

Session	Content
Session 1	Emotions located in various quadrants of the 2D model, such as high-arousal and negative-valence emotions
Session 2	Happiness and loneliness
Session 3	High-arousal and negative-valence emotions, such as stress and anxiety
Session 4	High-arousal emotions, such as perfectionism and anxiety (continue) and achievement

For the new loneliness component, in addition to discussing songs that evoke a sense of receiving empathy, psychoeducation will be provided on the positive effects of music sharing in building and strengthening emotional bonds in relationships [12]. The group sessions will offer a safe and pleasant environment where participants can talk about music, using this topic as a channel to foster new connections with fellow group members. In session 2, participants will be guided to discuss how they can apply this skill outside of the program to strengthen existing relationships and build new connections.

Participants are also expected to select music for themselves during the group sessions. These self-selected songs serve to evoke the target emotions for each session and support group discussions and experiential psychoeducation—specifically, increasing awareness of the bodily or physiological changes that accompany each emotion as elicited by music. As music appreciation is used as a tool to support psychoeducation about emotions, rather than being a goal in itself (ie, the program is not intended to enhance musical understanding), we believe that participant-selected songs are more appropriate than those prescribed or suggested by the program facilitators. This is also in line with literature supporting the benefits of using self-selected and familiar music [21]. As such, participants’ sharing of their self-selected music is incorporated as a core element in the sessions. The music submitted by participants spans a wide range of styles, reflecting their individual tastes, and includes Cantonese pop songs, classical music, movie soundtracks, and more.

Each session begins with a music check-in led by the music therapist, followed by participants listening to their self-selected music. This music listening and sharing segment takes approximately 15-20 minutes. Once the target emotions are evoked, the research assistant provides psychoeducation on the theme emotion and facilitates a group discussion on coping strategies. Around 10-15 minutes are allocated for this group discussion. Next, the music therapist guides participants through a core music activity designed to support reflection and reconnection with the theme emotion. Selected music activities include body scan, drawing, and lyric analysis. Each activity takes approximately 15 minutes. Each theme emotion is paired with its own specific psychoeducational content and corresponding music activity. The session concludes with a summary and debriefing provided to participants.

By the end of the last session, participants will be invited to complete the postquestionnaire. Individuals who complete both the prequestionnaire and postquestionnaire will receive a reimbursement of HK $200 (US $25.77) in cash. Reimbursement will be provided in person, and participants will be required to sign a receipt for record-keeping purposes. The intervention for the control group will be delivered after the final session of the experimental group and upon completion of the postquestionnaire by its participants. A flowchart outlining the project has been included in Multimedia Appendix 2.

### Outcome Measures

#### Overview

The following measures of emotion regulation (primary outcome), mood symptoms (secondary outcome), and loneliness (secondary outcome) will be administered pre- and postintervention via Qualtrics (Silver Lake Technology Management, L.L.C.).

#### Primary Outcome: Emotion Regulation

- The Emotion Regulation Questionnaire is a widely used measure with subscales assessing 2 styles of emotion regulation: cognitive reappraisal and expressive suppression. It has been translated and validated for use in Hong Kong (Cronbach α=0.80-0.83) [53]. A child and adolescent version of the scale has also been translated into Chinese [54]. Given that the primary focus of the Tuned In program is to encourage the outward expression of emotion through music, it is anticipated that changes will be observed in expressive suppression scores, but not necessarily in cognitive reappraisal scores, following the program.
- The Difficulties in Emotion Regulation Scale (DERS) is a more comprehensive measure that assesses emotional awareness, emotional acceptance, impulse control, and emotion regulation strategies among both adolescents and adults. This scale has also been validated for use with Chinese participants (Cronbach α=0.68-0.89) [55]. Given its comprehensive nature, the DERS is considered the primary outcome measure in this study.
- To measure confidence in managing emotions, items from the program evaluation tool used by Dingle and Carter [27], which explicitly asks participants to rate their self-confidence in managing both positive and negative emotions, will also be included.

#### Secondary Outcomes: Mood Symptoms And Loneliness

- The 21-item Depression Anxiety Stress Scale (DASS-21), which has been used with Chinese participants [56], will be used to measure mood symptoms. Recent research has demonstrated the scale’s validity for assessing emotional distress among young people in Hong Kong [57].
- The Hong Kong version of the 6-item De Jong Gierveld Loneliness Scale (Cronbach α=0.76) [58] will be used to assess loneliness.

### Additional Measure

We will use the Music USE (MUSE) Questionnaire [59] to assess each participant’s typical music listening habits, including whether they play a musical instrument and if they have received formal musical training. This scale was successfully used in a study involving 1318 adolescents in Hong Kong [34], making it a valuable tool for describing participants’ engagement with music.

For demographic information such as gender and socioeconomic status collected in the survey, tests will also be conducted across the 2 conditions to examine whether the results differ.

The effect of the intervention on reducing anhedonia will also be explored using the Chinese version of the Snaith-Hamilton Pleasure Scale [60]. Data collected from this scale will be used for future outcome analysis.

The Tuned In program’s acceptability among Hong Kong adolescents will be examined using a combination of quantitative and qualitative approaches. Satisfaction ratings will be collected from participants using a 7-point Likert scale. With “strongly disagree” at 1 point and “strongly agree” at 7 points, the ratings will capture the extent to which participants agree with the positive statements. The questions will address the perceived usefulness of the program, the level of interest in the program, the likelihood of recommending the program to other adolescents, and the likelihood of continuing to use music as an emotion regulation strategy in the future [25]. The aforementioned areas of investigation regarding the project’s acceptability are framed into 4 separate rating questions, all of which are included in the postintervention survey. The quantitative questions are provided in Multimedia Appendix 3.

In addition, we will include open-ended qualitative questions to gather feedback from participants on their experiences with the program and to collect suggestions for improvements for a future RCT. Written responses are expected from participants. As feasibility indicators, we will also record the recruitment rate and dropout rate for this trial. The qualitative questions are incorporated into the postintervention survey, to be completed by participants at the end of the final session.

To explore participants’ continued use of music for emotion regulation after the program, invitations to qualitative interviews will be sent at the 1-month follow-up. All participants who received the intervention will be invited. Open-ended and probing questions will be used to gather descriptive data on participants’ views of the program and their music listening habits. Data saturation is expected to be reached by the conclusion of the project. Please refer to Multimedia Appendix 3 for the interview and postintervention survey questions.

### Data Analysis

For the main outcome evaluation measures, mixed measures ANOVAs will be performed. The between-participants factor is group (intervention vs control), and the within-participants factor is time (pre- vs postintervention). Before the main analyses, independent samples *t* tests (2-tailed, paired) will be conducted on the baseline demographic variables to identify any significant differences between the 2 groups. Any variables that show statistically significant differences will be included as covariates in the subsequent main analyses. It is expected that the treatment group will demonstrate improved emotion regulation and reduced mood symptoms and loneliness following the music intervention, compared with the control group. A medium effect size is anticipated for the significant group × time interaction effect.

All verbal and written feedback will be analyzed using thematic analysis [61]. The qualitative analysis software NVivo (QSR International) will be used to assist with the process. Content analysis will be conducted following the transcription and coding of the data.

## Results

The successful funding outcome of this project was announced in February 2023. Participant recruitment began in January 2024, and as of September 16, 2024, a total of 316 responses had been received through Qualtrics. Upon submission of the questionnaire, all participants are promptly notified of its receipt. Those deemed eligible receive a basic information sheet, while individuals who are ineligible are provided with a list of supporting resources. Of the responses received, 89 participants were considered eligible for the program. The project is scheduled to conclude in August 2025, with results to be published thereafter.

## Discussion

### Expected Findings

This proposal focuses on examining the short-term impact of a music-based emotion regulation program for young people in Hong Kong. For this trial, we expect the intervention group to demonstrate improved emotion regulation and reduced mood symptoms and loneliness after completing the Tuned In program, compared with the control group. This expectation is based on previous successful trials of Tuned In conducted in Australia [27]. Furthermore, in line with the Transdiagnostic Emotion Vulnerabilities Model, enhanced emotion regulation may help alleviate a range of mood-related issues [7].

Qualitatively, we expect that participants will describe their reduced loneliness and enhanced mental well-being as being directly and indirectly related to music. For example, they may resonate with the music and experience a sense of being understood [12]. We also anticipate that participants will appreciate the use of self-selected music throughout the program [21]. In the future, we plan to conduct longitudinal studies to examine the medium- to long-term impacts of the intervention.

### Strengths

The study demonstrates notable strengths, including the involvement of 2 cofacilitators—a registered music therapist and a trained research assistant with a mental health background—ensuring both clinical expertise and research rigor in program delivery. Additionally, the inclusion of a new session component specifically targeting “loneliness” enhances the intervention by building upon and extending the established “Tuned In” program previously conducted in Australia, thereby addressing an important psychosocial issue with a tailored and evidence-informed approach.

### Limitations

As this study is a pilot RCT, the primary focus is on assessing the feasibility and effectiveness of the intervention. The long-term effects can be explored in future, larger-scale trials. It is acknowledged that the outcome measures used are self-reported. This method was chosen to capture participants’ subjective experiences and perceptions. Additionally, the study does not account for individual differences that may influence outcomes. Factors such as the impact of life events or levels of alexithymia may potentially influence the outcomes of the program and should be taken into account in future research. Subsequent studies could incorporate additional forms of assessment, such as physiological measures or reports from family members, to provide a more comprehensive and corroborated evaluation.

As coping through emotions mediates the relationship between resilience and youth well-being [62], equipping young people with adaptive strategies to manage stress and difficult emotions helps consolidate learning and builds greater internal resources and capacity. This, in turn, positively influences both their short- and long-term mental health. Healthy coping also acts as a protective factor for their future emotional well-being.

Although this project does not investigate the medium- to long-term effects of the intervention on youth mental health due to resource constraints, future research could adopt a longitudinal perspective to explore the factors that help sustain the positive outcomes. Future studies could also incorporate an active control design and examine the individual contribution of each component to the overall effectiveness of the program.

### Conclusions

This project aims to examine the short-term effects of a music-based emotion regulation program for young people in Hong Kong. We expect positive outcomes from this trial, and the preliminary results will inform the planning of future studies to better understand the underlying mechanisms and longer-term impacts.

From a practical perspective, the project will provide valuable insights into the use of music and psychoeducation in youth mental health work in Hong Kong. From a research and theoretical perspective, it may offer evidence regarding the effectiveness of combining music, psychoeducation, and group work in improving youth mental health. Building on the current project, future studies are encouraged to explore innovative ways to enhance the cost-effectiveness, outcome magnitude, reliability, and sustainability of the intervention.

## Appendix

Multimedia Appendix 1 Tuned In group session plan (session 5).

Multimedia Appendix 2 Project flowchart.

Multimedia Appendix 3 Survey and interview questions.

## Abbreviations

BRECVEMA: brain stem reflexes, rhythmic entrainment, evaluative conditioning, contagion (emotional contagion), visual imagery, episodic memory, musical expectancy, and aesthetic judgment
DASS-21: 21-item Depression Anxiety Stress Scale
DERS: Difficulties in Emotion Regulation Scale
GHQ-12: 12-item General Health Questionnaire
MUSE: music USE
RCT: randomized controlled trial
