# Supplementary material for: Using Music to Promote Hong Kong Young People’s Emotion Regulation and Reduce Their Mood Symptoms and Loneliness: Protocol for a Pilot Randomized Controlled Trial
Source: JMIR Res Protoc. 2025 Apr 16;14:e67764. doi: 10.2196/67764 (PMC12044316; doi:10.2196/67764)
Supplement: Multimedia Appendix 1 [file resprot_v14i1e67764_app1.docx]

Session 5:

Session rundown

| Program content/ Activities | Time allocation |
| --- | --- |
| Music check-in | 2 minutes |
| Introduction of session rundown & content. | 1 minutes |
| Homework review | 12 minutes |
| Activity 1: Facts about perfectionism | 7 minutes (introduction 2min + discussion 5min) |
| Activity 2: Facts about rumination, reflection, and its differences | 3-6 minutes |
| Activity 3: Encouragement/ acknowledgement | 10 minutes |
| Lyrics analysis for celebration | 10 minutes |
| Debriefing & Summary | 3 minutes |
