# Supplementary figures and images for: Using Music to Promote Hong Kong Young People’s Emotion Regulation and Reduce Their Mood Symptoms and Loneliness: Protocol for a Pilot Randomized Controlled Trial
Source: JMIR Res Protoc. 2025 Apr 16;14:e67764. doi: 10.2196/67764 (PMC12044316; doi:10.2196/67764)

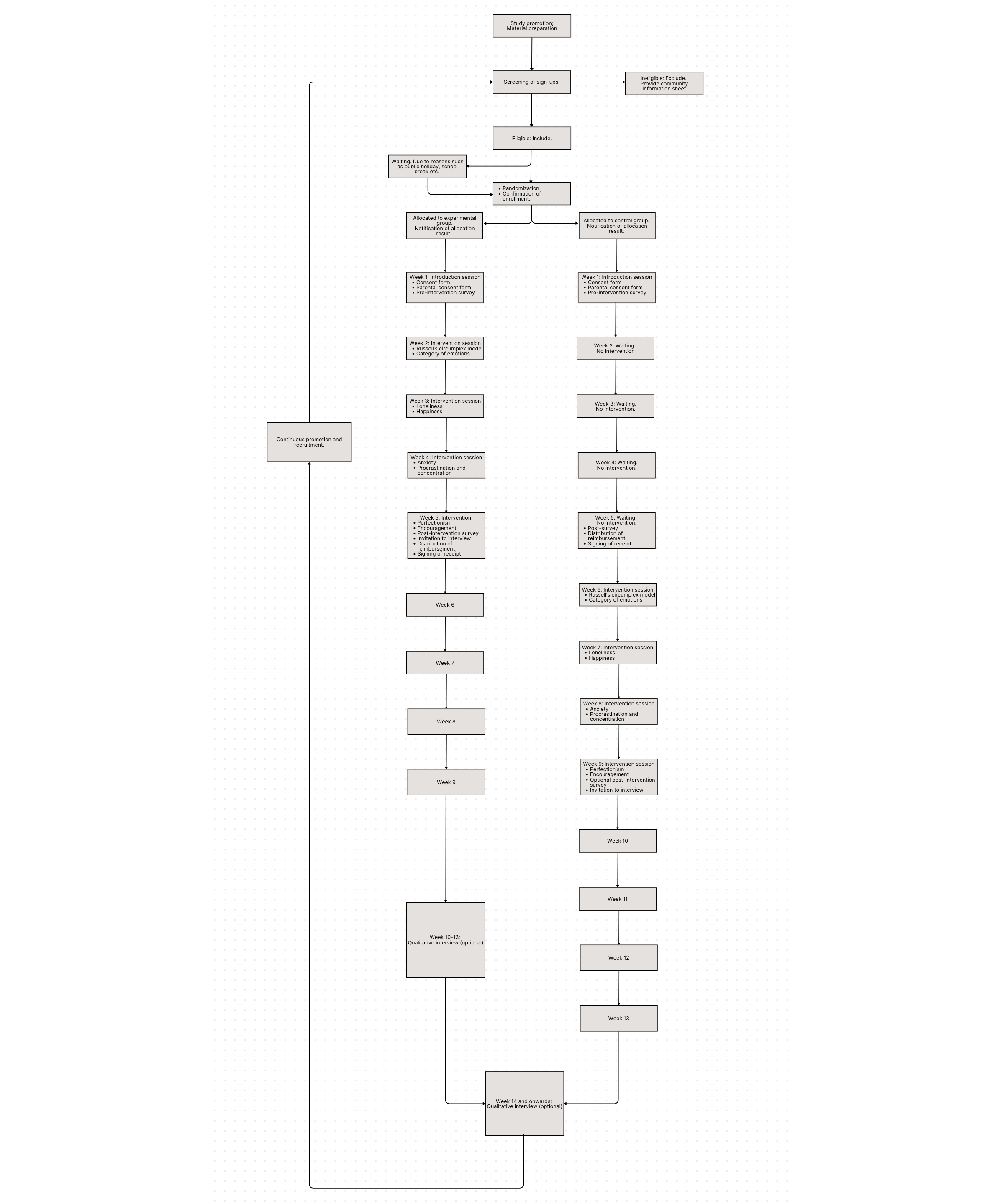

Supplement: Multimedia Appendix 2 [file resprot_v14i1e67764_app2.png]
