# Supplementary material for: Using Music to Promote Hong Kong Young People’s Emotion Regulation and Reduce Their Mood Symptoms and Loneliness: Protocol for a Pilot Randomized Controlled Trial
Source: JMIR Res Protoc. 2025 Apr 16;14:e67764. doi: 10.2196/67764 (PMC12044316; doi:10.2196/67764)
Supplement: Multimedia Appendix 3 [file resprot_v14i1e67764_app3.docx]

*Rating feedback*

Please rate to what extent do you agree with the following statement on a 7-point scale.

1 – Strongly disagree

7 – Strongly agree

- I consider Tuned In useful for me to manage my emotions.
- I consider Tuned In interesting and fun.
- I will continue to use music listening to manage my emotions.
- I will recommend this project to my friends.

*Qualitative written feedback*

- Please share your overall experience in the group.
- Which aspect of the group do you think can be improved? Please provide suggestions for our future improvement.

*Qualitative interview questions*

Takeaways and Learning:

1. Can you describe your music listening habits?
   1. What motivates you to listen to music?
   2. How would you describe your music taste?
   3. Under what context do you listen to music, and why?
2. How do you use music under different emotions?
   1. What types of music would you gravitate towards when you are feeling low/anxious/lonely? Why?
   2. What musical element or feature in a song helps you regulate the emotions? [e.g. tempo, melody, lyrics, familiarity, etc.]
   3. Can you give examples of how these musical elements or features help you regulate your emotions?
3. Do you remember what concepts you learned in "Tuned In”?
   1. Which ones do you consider important to you?
4. Do you find any of the 4 sessions memorable? Why?
5. Have your ways of listening to and engaging with music changed due to the program?
   1. How would you compare the use or engage with music before and after participating in the Tuned In program?
6. How has your understanding or ability to regulate emotions changed through the program?

Relevance and Application:

1. Were the scenarios and examples discussed in the program relatable to your own experiences?
2. What strategies from the program have you been able to apply in your real-life situations (e.g., breathing exercise, showering, seeking professional help, mindfulness, sports)?
   1. Do you find the strategy or technique from the program helpful during a productive task, like doing homework or studying?
   2. Do you find the strategy or technique from the program helpful during your daily life, such as relaxing or managing stress?
3. Which specific strategies have you used during or after participating in the program?
   1. Specifically, when you are feeling down / anxious/ lonely?
   2. What other strategies have you used besides music listening? Why did you choose those strategies over music listening?

Introduction Session:

1. What are your thoughts on the face-to-face introductory session before the start of the program?
2. Would you suggest any changes or additions to the introductory session?

Group Playlist:

1. What are your thoughts on the collaborative group playlist that was updated weekly during the program?
2. Did you find the group playlist to be a helpful resource?

Music-based Activities:

1. What are your thoughts on the three main music-based activities: guided imagery, bodily sensation, and lyrics sharing?
2. Which activity did you enjoy the most and why?
3. Have you been able to continue any of these activities in your daily life?
4. Do you have feedback on these activities?

Feedback and Recommendations:

1. What could be improved or changed for the next time this program is run?
2. Do you have any suggestions about the implementation, logistics, or facilitation of the program?
3. What did you think about doing this content in a group setting? (e.g. group dynamic, how willing they are to share things with group members?)
